# The influences of working memory representations on long-range regression in text reading: an eye-tracking study

**DOI:** 10.3389/fnhum.2014.00765

**Published:** 2014-09-29

**Authors:** Teppei Tanaka, Masashi Sugimoto, Yuki Tanida, Satoru Saito

**Affiliations:** ^1^Department of Cognitive Psychology in Education, Graduate School of Education, Kyoto UniversityYoshida-Honmachi, Sakyo-ku, Kyoto, Japan; ^2^The Japan Society for the Promotion of ScienceTokyo, Japan

**Keywords:** working memory, reading, eye-tracking, working memory capacity, reading span test, spatial span test

## Abstract

The present study investigated the relationship between verbal and visuospatial working memory (WM) capacity and long-range regression (i.e., word relocation) processes in reading. We analyzed eye movements during a “whodunit task”, in which readers were asked to answer a content question while original text was being presented. The eye movements were more efficient in relocating a target word when the target was at recency positions within the text than when it was at primacy positions. Furthermore, both verbal and visuospatial WM capacity partly predicted the efficiency of the initial long-range regression. The results indicate that WM representations have a strong influence at the first stage of long-range regression by driving the first saccade movement toward the correct target position, suggesting that there is a dynamic interaction between internal WM representations and external actions during text reading.

Reading involves dynamic interactions between internal representations and external actions. For example, when a reader encounters Sherlock Holmes saying, “the murderer is the man…” while reading a novel, identification of the person as well as his previously described utterances, attributes, and actions are important contributors to the enjoyment of the detective story. The reader may remember “the man” and easily identify the attributes that are central to the plot. In this case, internal representations in working memory (WM) are indispensable to reading comprehension, as suggested by a seminal study by Daneman and Carpenter (1980), who showed that individual differences in WM capacity (WMC) predicted successful remembering of the referent. Even with high WMC, when the story and the relationships among characters are complex, it may be difficult to remember previous descriptions. In such cases, readers need to relocate previous sentences to obtain the lost information. This process, called long-range regression, usually involves an external action, e.g., eye movements, but must be driven by internal representations, which direct the reader’s attention to the location where the lost information was presented. The external actions may in turn contribute to the (re)construction of internal representations by enabling readers to find the lost information that is important for understanding the story.

The primary purpose of this study was to explore the nature of the dynamic interaction between internal representations and external actions, which underpins the long-range regression process and is an important part of natural reading. It has already been shown that readers can identify (or relocate) the location of a word that was read previously, after reading text with a high level of accuracy (e.g., Rothkopf, 1971; Baccino and Pynte, 1994). These processes should be supported by internal representations generated from previously read text. In these cases, two types of representations could potentially underlie long-range regression in text reading: verbal representations and spatial representations.

Fischer (1999) suggested that when readers could use the spatial location of words, the location information could support word localization processes. However, when readers could not use spatial information due to its short-lived nature, they used nonspatial representations, such as information about a word’s temporal order, for the localization of words.

Some characteristics of readers’ word localization (relocation) abilities were examined by Rawson and Miyake (2002). In their experiment, participants were first asked to read 12 pages of text using a virtual book; they later engaged in an unexpected relocation task in which they were explicitly required to identify the locations (pages and lines) of some words in the text. The results showed that participants’ relocation accuracy was predicted by their verbal abilities, which were measured by a set of language tasks that included the reading span task (RST). However, there was no significant correlation between the relocation performance and visuospatial abilities, which were measured by a set of visual and spatial tasks, including a spatial WM task. The authors echo Fischer (1999) in suggesting that this difference can be explained by the faster decay of visuospatial representations compared to verbal representations (their relocation task was administered on a scale of minutes, rather than of seconds).

One of the notable features of Rawson and Miyake (2002) experiment is that all sentence letters were replaced by Xs during the relocation task (at the test phase). This method might have increased verbal cognitive load; consequently, only verbal (but not visuospatial) abilities showed correlation with relocation scores. In addition, Fischer (1999) pointing task and Rawson and Miyake (2002) relocation task were both off-line tasks rather than those requiring natural regression during reading. Thus, readers might execute their actions by using their explicit and/or episodic knowledge (see also Inhoff et al., 2005; Weger and Inhoff, 2007).

On the other hand, numerous studies demonstrate the beneficial effect of spatial representation on localization in reading (e.g., Kennedy and Murray, 1987; Kennedy, 1992; Kennedy et al., 2003). Furthermore, one eye-tracking study (Weger and Inhoff, 2007) revealed that both verbal and spatial representations play an important role in regression in reading sentences. Specifically, Weger and Inhoff argued that the first pass regression, which was the first regression to a particular word, was affected by spatial representations, whereas cumulative regressions, which were complementary regressions after the first pass regression, were supported by verbal representations. In their *within-line condition*, a sentence was presented in two separate lines; a target word, which appeared in the sentence, was aurally presented when readers’ eyes reached a predetermined sentence location. The predetermined position was at the right side of the sentence, in the same line in which the target word was visually presented. Participants were asked to read the sentence and required to regress their eyes on the target when the target was aurally presented. Two factors were manipulated: target distance (distant/close to the right edge of the sentence line) and target position (first line/second line). The results of Weger and Inhoff showed that the first pass regression was more accurate when the target was close; however, there was no influence of target position on the first pass regression. On the cumulative regressions, however, readers regressed their eyes for the target word in much more selective ways when the target was presented in the first line (i.e., verbal load was assumed to be low) than when the target was presented in the second line (i.e., the load was assumed to be high). In contrast, their *between-line condition* revealed the contribution of spatial knowledge during long-range regressions. In this condition, the predetermined region, in which the target word was aurally presented when readers’ eyes reached this location, was on the front of the second line of the sentence, and the target candidates were in the middle or at the end of the first line of the sentence. Thus, a target in the middle position was spatially close to, but verbally distant from, the predetermined region (a target in the end position was distant spatially, but close verbally). The result showed that the first pass regression was more spatially selective and more accurate when the target was spatially close (verbally distant) than spatially distant (verbally close). Results from their two conditions suggest that both spatial and verbal representations contribute to long-range regressions; however, their effectiveness is different in the two regression stages.

These previous studies used unique and powerful methods to examine the interaction between internal representations and external actions during reading, and successfully revealed the nature of representations employed for external actions (i.e., eye-movements). These experimental reading settings, however, are slightly different from the natural reading situation. For example, our long-range regressions are initiated spontaneously; thus, we are not explicitly required to regress to any words in response to an auditory word. A set of sentences (i.e., text), rather than a single sentence, are presented when reading a page of a book. Because they used single sentences in their study, analyses of eye-movements in Weger and Inhoff (2007) study were restricted to horizontal distances. But our real-world reading inevitably requires eye-movements in both horizontal and vertical directions.

In this study, we examined the nature and characteristics of representations that underpin long-range regression in text reading using an eye-tracking analysis, in which eye-movement accuracy was calculated based on both horizontal and vertical distances. In addition, we tested the influences of individual differences in verbal and visuospatial WM abilities on such processes. Our experimental task was based on a text comprehension task similar to a pronoun reference task (Daneman and Carpenter, 1980). For each trial, participants read a text, and were then asked to identify a particular person from the reading. Each text was one paragraph long and was presented on a single display page. This made the task duration quite short, which protected text representations (both verbal and visuospatial) from complete forgetting (due to decay or interference). The text paragraph remained available on the display while participants were answering the questions. This mimics a natural reading situation, in which all sentences are available even when readers forget some information that was read earlier. This also made participants execute spontaneous regressions.

Three types of eye movement indices were employed here. One was the number of fixations (NF), which is frequently used in eye-movement analyses. Fixations were defined as gaze points at which the reader’s eye movements stopped for more than 100 ms. If the reader’s regression process is inefficient, a larger NF should be counted between the presentation of the question and the participant’s response. The second index was the distance to the first fixation point while answering the question from the last fixation point on the question (i.e., regression size, RS). This index was used by Weger and Inhoff (2007) and is assumed to measure the spatial selectiveness, which reflects the usage of internal spatial representation in reading. The third index was introduced to estimate the preciseness of long-range regression—the distance to the target word (DT) from the first fixation point (Figure 1). These distances were calculated from *x*- and *y*-coordinates of the target word and of the first fixation point. We also recorded reaction time (RT) between the presentation of the question and the participant’s response.

**Figure 1:**
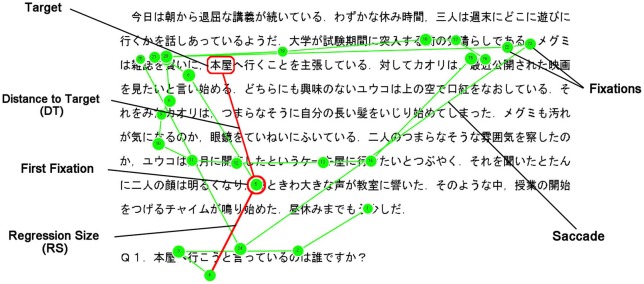
**An example of eye movements and indices on the whodunit task in Japanese**.

If readers execute their regression with spatial selectiveness induced by internal representations, a longer RS should be observed when the target is far from the question. If the readers know the location of the target word in the text precisely, the first saccade should directly move to the target from the question sentence. Consequently, DT should be shorter when the internal representations successfully guide the eye movements than when they do not. Our central assumptions here were that such internal (verbal and/or visuospatial) representations are supported by WM and that the efficiency of the long-range regression processes measured by the eye-tracking technique could be predicted by individuals’ WMC. Although it would be expected that WMC could predict the accuracy level of this task as well, the individual differences in accuracy would not be detected as a ceiling effect should be expected due to the presence of the target and the correct answer on the display while answering the questions.

## Methods

### Participants

A total of 51 undergraduate and graduate students who were all native Japanese speakers from Kyoto University participated in the experiment. We recruited participants only those who had good eyesight (without eyeglasses) or who were contact lens users. Therefore, all of them could participate in this study without glasses. The test session was divided over 2 days. The eye-tracking reading experiment was conducted on the first day, and two WM tasks took place on the second day. Participants provided written informed consent before the experiment on the first day, and received 1500 yen after the 2-day session.

### Stimuli and procedure

#### Reading task (whodunit task)

**Figure 2:**
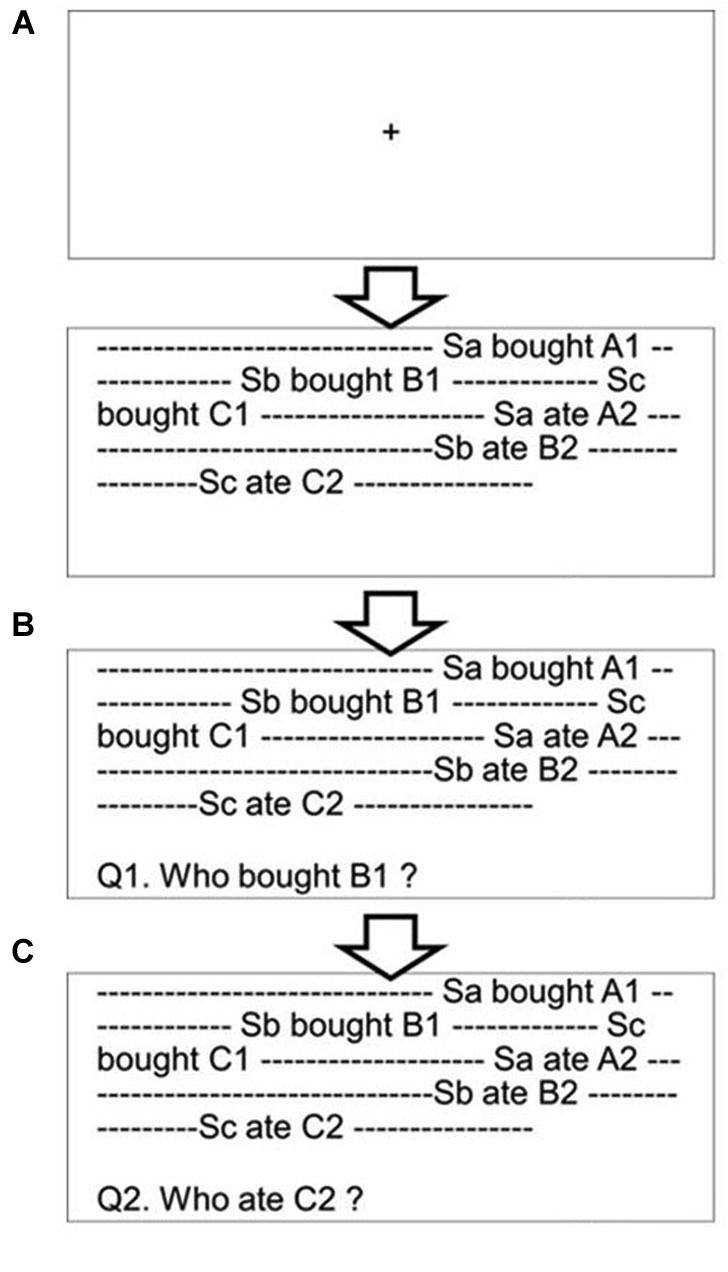
**The procedure of the whodunit task**. Sa, Sb, and Sc are character names. A1 and A2 are target actions of Sa (B1, B2, and C1, C2 are target actions of Sb and Sc, respectively). After participants read the text aloud **(A)**, the experimenter presented a question by pressing a key **(B)**. After the participants answered the question, the experimenter presented the next question by pressing a key **(C)**. The questions, the targets of which were randomly assigned to the top, middle, or bottom position, were presented three times in total.

Participants were required to read aloud a text presented on a PC screen. Although the reading aloud procedure did not provide a perfect natural reading situation, this setting had produced several benefits for our experiment. First was that we could easily monitor the participants’ text reading. Second was that the reading aloud setting inhibited readers’ articulatory rehearsal during reading phase. Moreover, this procedure could prevent readers’ unregulated regressions during the reading phase, securing eye-tracking immediately after the text reading. One text contained one paragraph consisting of a mean of 348 (*SD* = 2.43) Japanese kanji and kana letters and of 11.55 (*SD* = 1.01) sentences (Figure 2A). Most of the sentences held an SOV structure common in Japanese. Letters (Gothic font, font size 18 pt.) were presented in black color on a white screen. Sentence text (9 lines) was displayed with double spacing and there were triple line spaces above and beneath the text region.

Three characters with female Japanese names, i.e., Kaori, Megumi, and Yuuko, appeared in every text (trial), and their characteristics, activities, and/or thoughts were described in a story. In total, six descriptions were presented in each text (two for each character). Below, we refer to these descriptions as the target. Each text started with one or a few sentence(s) that described a situation of the story (i.e., the number of sentences was 1, 2, or 3) and that was/were followed by the six target descriptions. The first three target descriptions (one for each character) and the last three target descriptions (one for each character) consisted of three to six sentences, respectively. In the majority of stories (16 among 18 trials), one or two sentences was/were presented between the third and fourth target descriptions in order to describe a situation for the last three target descriptions. The final one or two sentence(s) finished the story. When participants finished reading a text aloud, the experimenter pressed a key to display a question under the text (Figure 2B). The question asked who was responsible for performing the act named in the target word (e.g., *“Who ordered a cup of coffee?”*); we called this a *“whodunit question”* and in this case, the target word is “coffee”. All target words were a noun. Participants were asked to answer the question by pressing one of three keys, each of which was assigned to one of the three female names. The same three female names appeared in all trials. This setting allowed participants to learn the correspondence between names and keys perfectly. Thus, they did not need to look down at the keyboard when they made their response. Participants were told to answer the question as quickly and as accurately as possible, and they were permitted to reread the original text before giving their responses, as the text was still present in front of them. Eye movements were recorded between the presentation of the question and each participant’s response. Three questions were asked in each trial. After participants answered the first question, the next question was presented immediately (Figure 2C). The three questions were derived from the top position (the first or second targets; presented on lines between 2 and 4), middle position (the third or fourth targets; presented on lines between 4 and 7), and bottom position (the fifth or sixth targets; presented on lines between 6 and 8) within a text, respectively. The presentation order of the three questions was randomized for each trial. Before and during two practice trials, participants received instructions about the experimental procedure and learned the associations between the three character names and their respective key positions. 18 test trials were divided into three blocks, and at the beginning of each block, eye movements were recalibrated. The order of the three blocks was randomized.

#### WM tasks

We employed the Japanese version of the RST (Osaka, 2002) for the measurement of verbal WMC, and the spatial span task (SST; Maehara and Saito, 2007) for the measurement of spatial WMC. We used RST stimuli from Osaka (2002), and SST stimuli from Maehara and Saito (2007). In both WM tasks, set size was from 2 to 5 and there were three trials in each set size. Thus, the maximum score on both the RST and SST was 42 points. The order of the 12 trials was randomized. The RST required participants to read aloud a series of unrelated sentences (presented one by one), in which a to-be-remembered word was underlined with a red line, and asked to recall the set of target words in the presented order (e.g., in set size three, participants were asked to read aloud three sentences and asked to recall three target words in total). The SST required participants to engage in a spatial processing task (same-different judgment on two meaningless shapes) and a spatial memory task (remembering the position of a dot in 4 × 4 matrices) alternatively. During the recall phase, participants were asked to recall the dot positions in the presented order by writing them down (e.g., in set size three, participants repeated the “spatial judgment—dot memory” cycle three times and were asked to recall the three dot positions by writing them down). Performance of RST and SST was evaluated by the total number of correctly recalled items, which were target words for RST and dots in matrices for SST, at the correct serial position (see Friedman and Miyake, 2005).

### Apparatus

A Tobii T120 eye-tracker was used for recording eye movement during the reading task. Participants were not fixed by chinrest or bitebar, as the Tobii T120 can capture and record eye movement in a natural reading state. The distance between participants and display was approximately 40 cm. The screen resolution was 1280 × 1024 pixels (17″). The reading task was controlled by Tobii Studio 1.7.2. Both RST and SST were administered on another PC (screen resolution was 1920 × 1200, 23″) and controlled by Super Lab., version 4.0.

## Results

Data from 17 females and 23 males (ages ranged from 18 to 29 years) were included in the following analyses. Data from other participants were not analyzed because of eye-tracking failure (6 participants), inadequate reading (e.g., skipped some words in text reading; 2 participants), very low average correct answer rates (not different from or lower than the chance level; 2 participants), and experimenter’s failure (1 participant).

The average score for RST was 26.65 (*SD* = 5.01), and that for SST was 28.83 (*SD* = 6.48). A significant correlation between RST and SST (*r*_(40)_ = 0.64, *p* < 0.01) was found. The average total number of correct responses in the whodunit task was 52.65 (*SD* = 1.87) for 54 questions (three questions in each of 18 trials). Our participants frequently looked back to the text region after the presentation of the whodunit question (45.28 times in average, *SD* = 8.87) and most of their responses were correct in those cases (*M* = 44.33, *SD* = 9.13). Even when they did not look back (i.e., without regression; 8.73 times in average, *SD* = 8.87), our participants responded correctly in the most of the cases (*M* = 8.33, *SD* = 8.78). As predicted, the accuracy of the whodunit task was almost at a ceiling and the majority of the responses were accompanied by a long-range regression. In the present study, we analyzed RT and three types of eye-movement measures, which were recorded between the presentation of the question and the participant’s response only when participants answered correctly with regressions.

The RT, NF, RS, and DT were subjected to an analysis of variance (ANOVA) with the within-subjects variable of target position (three levels: top, middle, and bottom).^1^ Descriptive statistics from these measures are presented in Table 1. A significant main effect of target position was found for all indices, RT: *F*_(2,78)_ = 16.24, *p* < 0.01, *MSE* = 75467.50, *η*^2^ = 0.29; NF: *F*_(2,78)_ = 17.93, *p* < 0.01, *MSE* = 1.45, *η*^2^ = 0.31; RS: *F*_(2,78)_ = 22.39, *p* < 0.01, *MSE* = 2707.44, *η*^2^ = 0.36; DT: *F*_(2,78)_ = 59.74, *p* < 0.01, *MSE* = 2661.61, *η*^2^ = 0.60. Subsidiary analyses (with Bonferroni corrections) indicated that the longest RT, the largest NF, and the longest RS and DT occurred when target words were at the top position. In contrast, the shortest RT, the least NF, and the shortest RS and DT occurred when target words were at the bottom position. There were significant differences between top and middle positions (*p* < 0.05 (RT), *p* < 0.05 (NF), *p* < 0.01 (DT)), top and bottom positions (all *p*s < 0.01), middle and bottom positions (all *p*s < 0.01). There were significant differences between top/middle positions and bottom position on the RS index (both *p*s < 0.01); however, there was no significant difference between top and middle positions on this index (*p* = 0.14, *n.s*.).

**Table 1:** **Descriptive statistics of RT, NF, RS, and DT on each target position**.

	Reaction time	Number of fixations	Regression size	Distance to target
Top	3000 ms (530)	11.20 (1.93)	541.25 pixel (84.97)	417.98 pixel (50.54)
Middle	2808 ms (486)	10.36 (2.45)	516.72 pixel (92.48)	342.07 pixel (58.59)
Bottom	2650 ms (488)	9.58 (2.15)	464.98 pixel (97.17)	292.82 pixel (60.54)

*Note. Standard deviations are in parentheses*.

Table 2 shows correlations among the three eye-movement measures (i.e., NF, RS, and DT) at each target position, the two WM measures and RT. NF was not correlated with RST and SST scores at any position. However, DT for the middle-position targets showed significant negative correlations with RST and SST scores, although DT for other positions did not. A subsequent partial correlation analysis revealed that when SST performance was controlled, the partial correlation between DT for the middle position and RST scores dropped to a non-significant level, *r*_(37)_ = −0.21, *p* = 0.18. After controlling for RST performance, the partial correlation between DT for the middle position and SST scores also decreased to a non-significant level, *r*_(37)_ = −0.21, *p* = 0.19. As Table 2 shows, RT and NF at each target position were positively correlated. DT showed a significant correlation with NF and RS only at the bottom position but not at the other target positions. The mean text reading time was 55.01 s (*SD* = 61.71), and no significant correlations were found between RT and RST performance in any positions (*r*_(40)_ = −0.14, *p* = 0.36 (top); *r*_(40)_ = −0.25, *p* = 0.11 (middle); *r*_(40)_ = −0.26, *p* = 0.09 (bottom)) and between RT and SST performance (*r*_(40)_ = 0.17, *p* = 0.28 (top); *r*_(40)_ = −0.01, *p* = 0.94 (middle); *r*_(40)_ = −0.15, *p* = 0.34 (bottom)).

**Table 2:** **Correlation between eye-movement measures (NF, RS and DT) at each target position and reaction time at each position and two working memory task scores**.

	NF			RS			DT		
	Top	Middle	Bottom	Top	Middle	Bottom	Top	Middle	Bottom
RT at each position	0.78**	0.78**	0.74**	−0.03	−0.01	−0.04	−0.11	0.16	0.05
NF at each position	—	—	—	0.06	0.26	0.21	0.00	0.22	0.39*
RS at each position	—	—	—	—	—	—	−0.16	−0.19	0.65**
RST	−0.18	−0.20	−0.23	0.14	0.10	0.23	−0.08	−0.41**	0.11
SST	0.05	−0.13	−0.21	0.01	−0.13	−0.04	−0.25	−0.41**	0.00

*Note. RT = reaction time, NF = number of fixations, RS = regression size, DT = distance to target, RST = reading span task, SST = spatial span task. Significant correlations were not found between RT and RST performance (rs_*(40)*_ = −0.14, −0.25, and −0.26 for the top, middle, and bottom, respectively) and SST (r_*(40)*_ = 0.17, −0.01, and −0.15 for the top, middle, and bottom, respectively)*.

** p < 0.01, * p < 0.05.

## Discussion

The current study reconfirms spatial selectiveness during long-range regressions when reading text and offers two sets of novel findings. Analysis of RS shows that RS was smaller when the target word was at a bottom position than at a top or middle position. This result suggests that when readers execute long-range regression, their eyes regress spatially selectively rather than randomly landing (Weger and Inhoff, 2007). One of the novel findings is the target position effect observed in three measures (RT, NF, and DT) of our text-reading task; that is, the least efficient regression performance for the top-position targets and the better performance for the bottom-position targets. The other new finding is a specific correlational pattern between the eye-movement data and the WM scores.

The former finding indicates that it was more difficult for participants to regress accurately and efficiently when target words were at the top position than when they were at the middle or bottom position. This suggests that the availability of text/target representations decreases, either over time or due to interference from subsequent reading activities. Thus, the effect seems to reflect the characteristics of forgetting in WM (Towse et al., 1998; Maehara and Saito, 2007; Barrouillet and Camos, 2012; Oberauer et al., 2012). Although the current dataset cannot specify the mechanisms of forgetting in WM, it is certainly consistent with the idea that WM is involved in our text-reading task.

The results from the correlation analyses provide an additional clue to understanding the nature of WM representations/processes in the text-reading and long-range regressions. One of the crucial results in this study is the correlation between DT and WM scores. Both verbal and visuospatial WM performance predicted DT for the middle-position targets. Furthermore, the partial correlation analyses indicated that verbal and visuospatial WM shared variance in predicting DT at the middle position. Although DT showed this correlational pattern with WM scores, other eye-movement measures (NF and RS) were not correlated with WM measures. Note that as Table 2 shows, this NF measure was correlated with RT at all three target positions, but RS and DT were not, indicating that NF and RS/DT reflect different cognitive processes, although both are eye-movement measures.

NF reflects the visual search process while participants are looking for the target word after the first saccade, and increasing NF leads to the linear increase in RT. Because our text-reading task left all text information on the display even after the question was presented, the search process might be similar to that required in a typical visual search experiment (e.g., Treisman and Gelade, 1980). Although the efficiency of the visual search is sometimes affected by WMC in a very specific situation (e.g., Poole and Kane, 2009), it has been widely accepted that WM scores do not modulate the patterns of typical visual search processes (Kane et al., 2006). Therefore, the lack of correlation between NF (and RT) and WM scores is consistent with findings from the previous WM literature.

RS and DT do not reflect eye movements during visual search processes, but do reflect the accuracy of the first saccade before starting the visual search. On the first saccade for the long-range regression, participants cannot use the external text information because that saccade starts before they look back over the text. Instead, they have to rely on internal text representations (either rough gist or detailed representations) in guiding their first saccade eye movement. We assume that RS might reflect gist text information, which can provide rough information regarding the target position (e.g., top, middle, or bottom). The results of RS reconfirmed this spatial selectivity of long-range regression (Weger and Inhoff, 2007). On the other hand, DT might reflect the precise information regarding the target location, which could be the internal detailed representations (verbal, visuospatial, or both) that are supported by WM. Thus, the availability of such detailed representations may reflect individual differences in WMC, predicting the correlation between DT and WM scores. It is assumed that when readers could not use detailed representations for initial regression, they might use a rough gist. We observed no correlations between RS and WM scores. This result is consistent with the assumption that RS might reflect gist text information but not reflect individual differences in WMC. One may note that there is a significant correlation between RS and DT at the bottom position and that this correlation seems to be strange if the two indices are reflecting different aspects of internal representations. Although the most of RSs and DTs for targets at the bottom positions were very short, some of the first saccades for those targets occasionally went beyond the bottom and arrived at the middle or at the top positions erroneously. In these cases, RS and DT were both very long, leading to the strong positive correlation between the two indices.

In summary, long-range regression entails at least two steps with differential scaffolds. The first step, in which readers could not use external text information, might be guided by readers’ internal representations. In this step, when readers have detailed representations (e.g., detailed word position information) they could regress accurately near the target (reflected in DT). Even when they have only rough gist representations, however, they could regress roughly around the target (reflected in RS). The second step is underpinned by external text information, and its operation is measured by NF and RT. The idea that WMC seems to affect only the first process by holding the internal text representations can generally explain the presence, and the absence, of correlations between WMC and other measures. Here, two unresolved questions remain. First, why did we find the correlation between DT and WM scores only for the middle target position? Second, what is the nature of the internal representations (or mechanisms) that mediate correlations among DT, verbal WMC, and visuospatial WMC?

Why should we find a correlation between DT and WM scores only for the middle target position but not for the top and the bottom target positions? One approach to this issue can be provided by the notion of the capacity limitation of WM. Cowan (2001, 2010) indicated that our ability to hold bound objects in WM is limited to a certain number, i.e., four. The present text-reading task had six target actions. As we discussed later, participants would be more likely to bind the verbal and spatial information of the targets during text reading (a recent estimation of the capacity limit of verbal-spatial bound objects is said to be three; Langerock et al., 2014). If we accept that the bound representations for both verbal and spatial aspects operate as bound objects, then we can assume that only the last three or four target actions (one or two middle positions and two bottom positions) were within WM. The accessibility of these four targets was higher than that of the first two targets (i.e., those in the top position), which had already gone to long-term memory. Within WM, due to decay or representational interference, the targets in the middle positions, at the boundary of the capacity limit, may have had degraded representational quality that was different from that of the targets in the bottom positions. It is likely that the degree of representational degradation would show large inter-individual variations based on WM functioning. Therefore, it is expected that the individual differences in WM scores should have an impact on relocation processes measured by DT predominantly at the middle target positions. There might be another possible explanation of the significant correlations at the middle positions. The majority of our text stories used in this study consisted of two situations of which the boundaries located between the third and fourth target descriptions. As a shift of a mental model from one to another could potentially require additional resources of WM, it might be the case that, due to the WM load, the targets in the middle positions might have had degraded representations compared to that of the targets at the top and bottom positions. This view also predicts that the individual differences in WM scores should have an impact on relocation processes at the middle target positions. Although these two views provide speculative explanations, both expect that the individual differences in WM should exhibit its predictive power at the middle positions.

Then, what is the nature of the internal representations (or mechanisms) that mediate correlations among DT, verbal WMC, and visuospatial WMC? The correlational analyses suggest that the internal representations and the representational mechanisms that can guide the first regression may be domain-general. Some shared variances between verbal and visuospatial WM are associated with individual differences in DT. To accomplish the relocation smoothly, participants should return their eyes to a location near the target word. For this action, not only verbal information from the context, but also spatial information—such as the estimated position of the target—would be important scaffolds. In this situation, there were at least two possible ways that verbal and visuospatial WM could have had an impact on relocation processes. The first is that two WM domains may affect relocation independently. Another is that a common domain-general component of WM (i.e., executive attention; see also Kane et al., 2006) underpins long-range regression processes. The partial correlation analysis suggests that the latter assumption seems to be the correct one. A study by Cowan et al. (2006) that examined verbal-spatial associations may support this assumption. In their study, participants were presented with a series of names sequentially, one at a time, each located in one of a group of schematic houses at different locations. Participants were then required to remember the names and their locations (houses). Adults could perform this task efficiently by remembering the sequence of the names and that of the locations separately and then combining these modality-dependent memories in a recall phase. Third-grade children, however, did not employ this efficient strategy. Instead, they seemed to combine the names and the houses at an encoding phase and try to maintain these bound representations throughout the experiment. The adult participants did not use this binding strategy because it requires high attentional demands. However, the results showed that the adult participants were forced to use the binding strategy when they performed the task under articulatory suppression, which is assumed to prevent participants from using articulatory rehearsal for the retention of verbal materials (e.g., a sequence of the names).

In our reading task, both verbal and visuospatial aspects of the task-related information were important to accomplish the task. Moreover, the task required participants’ oral reading, which could potentially prevent them from employing articulatory rehearsal during performance of the whodunit task. Thus, it is possible that our participants may have tried to use the binding strategy, attempting to bind the potential target words and their locations while reading the text. Although the precise mechanisms of this binding function have not been specified, this function likely requires domain-general resources involving attentional control mechanisms for combining two different modality-dependent sets of representations in WM. It is assumed that this type of bound representation of verbal and visuospatial information might be used in guiding the first regression movement from the whodunit question. Therefore, the quality or availability of the bound representations could potentially have an influence on the efficiency of the relocation processes, reflected in DT; consequently, individual differences in the quality of the bound representations might affect the variability of DT.

We would like to note that the bound representations might be not solely based on presented information, particularly in the case of text reading. Recent studies (van Dijck and Fias, 2011; van Dijck et al., 2013) reported that each word in a temporal sequence of words have a respective spatial value (e.g., the first one has “left” and the last one has “right”) in WM and the words in the middle of the sequence have less spatial value. This finding indicates that words presented in a text receive spatial information not only from presented positions but also from presented order. The bound representations might be influenced by the position and order information. If this is the case, the bound representations for targets at the middle position would be more fragile due to the weaker spatial value, thus strongly exhibit individual differences of WM as reported in this paper. However, it is not clear whether spatial information from presented order includes the top—bottom contrast. This issue must be examined in future studies.

In the present study, we found a dynamic interaction between internal WM representations and external actions during text reading. Specifically, such an interaction occurs in long-range regression processes, demonstrating that internal WM representations have a strong influence at the first stage of the relocation process by driving the first regression movement toward the correct target position. The influence of WMC seems to operate through the domain-general WM system, perhaps through attentional control mechanisms for binding verbal and spatial information. By using eye-movement analyses and the whodunit task, we were able to build upon developments in previous work to produce a more natural reading situation. The micro activities, such as eye movements, that are usually difficult to capture were clearly affected by individual differences in WMC, and the effects are elucidated by the eye-tracking technique.

## Conflict of interest statement

The authors declare that the research was conducted in the absence of any commercial or financial relationships that could be construed as a potential conflict of interest.

## Footnotes

^1^Kurtosis and skewness of four indices in each positions were between −1 and 1, except RT in top position (kurtosis was 3.93, skewness was 1.17) and NF in middle position (kurtosis was 2.11, skewness was 1.15). We conducted ANOVAs on log transformed RTs and square-root transformed NFs in all positions. The same patterns (including subsidiary analyses) as ANOVAs without transformations were obtained. We reported analyses of the non-transformed data for all four indices.
